# Community-Partnered Development of Behavioral Economic Incentives in a Pre-exposure Prophylaxis Intervention for Transgender and Nonbinary Clients in Los Angeles

**DOI:** 10.21203/rs.3.rs-7654557/v1

**Published:** 2025-10-29

**Authors:** Mika Baumgardner, Carrie L. Nacht, Kimberly Ling Murtaugh, Risa Flynn, Chloe Opalo, Alex R. Dopp, Erik D. Storholm

**Affiliations:** Los Angeles LGBT Center; University of California, San Diego; University of California Los Angeles; Los Angeles LGBT Center; Los Angeles LGBT Center; RAND Corporation; San Diego State University

**Keywords:** pre-exposure prophylaxis, transgender and nonbinary, LGBT health, HIV, behavioral economics, economic incentives, community-engaged research

## Abstract

**Background:**

This article describes our community-engaged process of incorporating culturally relevant, non-monetary, chance-based incentives into a transgender and gender nonbinary (TGNB) oriented pre-exposure prophylaxis (PrEP) intervention called *PrEP Well*.

**Methods:**

*PrEP Well*, a single-group longitudinal observational study design, was developed as a community-led, multicomponent HIV prevention intervention that combines peer navigation and fixed monetary incentives for research activities in a TGNB community health center. Recruitment for *PrEP Well* began in April 2022. We added prize-based behavioral economic incentives to *PrEP Well* on April 25, 2023. These prizes reflect the TGNB community’s interests through input from a transgender community advisory board, surveys of health center clients, and partnerships with local TGNB-owned businesses. Data are drawn from program administrative records, surveys, and qualitative interviews with program recipients. Comparisons between prize selection were assessed via Chi-square tests. Qualitative interviews were analyzed via inductive thematic analysis.

**Results:**

Before implementation, survey respondents found additional incentives for participation in *PrEP Well* were highly acceptable. After the incentives were implemented in *PrEP Well*, qualitative data from exit interviews highlighted the motivational impact of combining a fun, chance-based incentive model with the empowering ability to choose from a variety of trans affirming non-monetary incentives.

**Conclusions:**

Our community-partnered approach integrated behavioral economic incentives into a TGNB-oriented PrEP intervention. The participants found the additional community-relevant prize-based incentives to be acceptable and motivating. Future research should explore the immediate and long-term outcomes of this approach, and its scalability with diverse TGNB populations, to promote equity in HIV outcomes.

## Background

HIV continues to disproportionately affect transgender and gender nonbinary (TGNB) individuals.^1,2^ Pre-exposure prophylaxis (PrEP) medication is a highly effective method of HIV prevention,^3–8^ but TGNB people are underrepresented across the PrEP services continuum.^2,9^ as well as in research on PrEP uptake and adherence.^10–12^ TGNB people experience unique barriers to healthcare access, often shaped by stigma, discrimination, lack of culturally competent care, and other socioeconomic and structural challenges.^10,13–15^

Although there is a pressing need to develop and implement culturally competent PrEP interventions tailored to TGNB communities, health services often do not support TGNB needs. A 2022 scoping review revealed that many health interventions grouped TGNB individuals with broader lesbian, gay, bisexual, transgender, and queer (LGBTQ) categories, lacked TGNB input in their design, and did not adequately address intersectional barriers.^16^ Several studies have demonstrated that interventions with promising results among broad LGBTQ populations can be ineffective for TGNB recipients.^16–18^ Interventions are more effective when they elicit feedback from TGNB communities at all levels of design and implementation,^16,19^ including interventions targeting HIV prevention and care access in TGNB communities.^19–24^

Designing incentive systems tailored to TGNB community is key to motivating health behaviors and reducing barriers for the community. Financial incentives have been shown to motivate participation in randomized controlled trials to promote a variety of health behaviors including smoking cessation, substance use abstinence, and adherence to HIV medications.^25^ Behavioral economic interventions use incentives designed around decision processes and individual preferences that shape behavior.

Studies have shown that chance-based financial incentives can be powerful motivators for HIV prevention and other health related behavioral changes.^26–29^ Studies suggest that regardless of the financial amount offered, incentives that are fun and focus on the recipient’s ingroup community and identity can lead to long term behavioral change.^27, 30–33^ Moreover, one study reported that simply giving participants the ability to make a decision about a hypothetical prize empowered them by increasing feelings of control and joy.^34^

Creating an incentive system that uses behavioral economics to engage TGNB individuals with culturally specific and empowering incentives can enhance these perceptions of fun, community identity, and control to sustainably increase HIV prevention behaviors. Several studies applied behavioral economic incentives to motivate HIV-related health behaviors among cisgender people^26–29,35,36^, but there is little comparable work among TGNB populations. Nevertheless, the limited data are promising.

One study with HIV-positive transgender women of color that offered unlimited peer health navigation and escalating monetary incentives for behavioral and biomedical milestones reported increases in HIV care participation across up to 3 years of program participation.^37^ Another study revealed that chance-based incentives in addition to informational text messages resulted in significant improvements in HIV knowledge compared to a control group that received informational text messages only. Although the sample was mostly Latinx sexual minority men, approximately one-third of the participants were Latinx transgender women (*n* = 66).^27^

These studies all used monetary incentives and provided limited information about how the incentives were developed for their TGNB recipients. As is largely the case in related areas of research, transgender women have also been the focus of relevant work to date. None of these studies created non-monetary incentives tailored to the needs of the entire TGNB community. In summary, the use of tailored behavioral economic incentives for HIV prevention is understudied with TGNB communities, limiting the design of prevention interventions that support this population. The purpose of this paper is to describe a community-engaged process that involves tailoring a behavioral economic prize-based incentive structure to increase PrEP engagement and adherence among TGNB individuals.

## Methods

### Study setting and design

We partnered with a TGNB-serving community organization to conduct this study as a component of their ongoing *PrEP Well* program. *PrEP Well* is a peer navigation intervention that connects participants with gender-affirming PrEP services at a TGNB-serving organization.^38^ The organization is a federally qualified health center in Los Angeles, California that offers many services, including primary health care, HIV testing and treatment, legal and employment services, etc.^39^ Full details on the community-led design and implementation of *PrEP Well* have been described elsewhere.^38^

The design of *PrEP Well* was informed by TGNB community members and staff.^40^ Briefly, *PrEP Well* aimed to bring comprehensive community-led PrEP services onsite to the TGNB-serving organization, where anyone who identifies as TGNB that is 18 years or older can enroll. *PrEP Well* includes peer-led enrollment and navigation along the PrEP care continuum and 30-day and 90-day follow-up visits. *PrEP Well* began enrolling participants in August, 2022, and recruitment is still ongoing.

*PrEP Well* provides financial incentives to participants for attending each visit, regardless of their engagement with PrEP ($50 for baseline, $75 for 30-day follow-up, $100 for 90-day follow-up) to collect data on engagement barriers. The participants expressed a need for additional incentives. Therefore, the current study sought to develop and implement a supplemental behavioral economic incentive system that would motivate participants to increase PrEP engagement and adherence.

Development of the behavioral economic prize-based incentive system began in January, 2023, and the system was implemented on April 25, 2023. The demographic data of the participants who received the newly implemented incentives were collected from April 25, 2023 through January 1, 2025 (Table 1).

**Figure 1:**
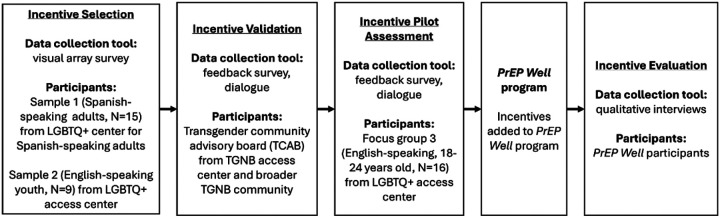
Multi-step evaluation to community-engaged incentive selection, assessment, and evaluation

We use a descriptive approach drawing from multiple data sources (surveys, project administrative records, interviews) to illustrate the community-engaged design approach (Fig. 1).

### Creating the Prize Wheel and Incentive selection

The research team drafted procedures describing how incentives would be implemented in *PrEP Well* (Table 2). The participants could receive up to 3 spins per visit (9 total) over the study duration. After participants spun the wheel, they could browse a catalog of prize options and select their desired prize.

To frame the incentive exchange as a fun game with themes of TGNB empowerment, the incentive levels reflect the colors of the transgender pride flag: pink (70% likelihood, average cost $0.88, retail value $1–6), blue (25% likelihood, average cost $5.25, retail value $10-$30), and silver (5% likelihood, average cost $51.43, retail value $50–100). The average cost of items reflects significant savings in bulk discount purchases and other cost-saving techniques. Based on behavioral economic theory about the motivational advantages of identity-related incentives,^30,33^ we engaged community members to select prizes that would be perceived as fun, valuable, and tailored to the TGNB community. First, we generated a range of prize options with input from TGNB staff of the community organization. The research team included prizes sourced from TGNB-owned businesses to authentically build trust, representation, and resilience with the community through economic empowerment.

To engage TGNB-owned businesses, the research team utilized transowned.co, an online directory of verified TGNB-owned businesses. The team contacted businesses to verify they were comfortable being denoted as “Trans Owned” within our study context. Conversations with some TGNB-owned businesses led to referrals to other businesses. We also commissioned a local TGNB artist to create original art and prizes. Additionally, some prizes were purchased through digital storefronts such as Amazon.com.

To assess the incentives selected as prizes for *PrEP Well* participants, we created a visual array survey (i.e., with images of each item to maximize understandability). In February, 2023, we distributed this survey to Focus Groups 1 and 2, two convenience samples composed of clients at two LGBTQ-serving settings to guide incentive selection for *PrEP Well* participants. There was no incentive for survey takers.

We first sought feedback on our incentive procedures from a transgender community advisory board in February, 2023; dinner and small monetary incentives were provided as compensation for participation. Second, we sought feedback from a convenience sample of clients at an LGBTQ youth access center, who were invited to spin the prize wheel and win a prize of their choice in exchange for their participation. At the end of both presentations, board members and youth completed a qualitative feedback survey asking what they thought about *PrEP Well*, the prize wheel, and the prizes offered. The participants were also invited to participate verbally during these engagements. After implementing the wheel, we totaled how many participants chose each prize, from April 25, 2023 through January 1, 2025.

### Qualitative interviews

All participants in *PrEP Well* were eligible to participate in exit interviews. The interview methodology was developed for *PrEP Well* and is described elsewhere, in a currently unpublished manuscript.^41^

From September 2023 to August 2024, semi-structured interviews were conducted by the *PrEP Well* peer navigator, who asked about facilitators, barriers, and suggested improvements for *PrEP Well*. The interviews were conducted in English or Spanish, transcribed, translated to English if in Spanish, and de-identified. For the current study, we drew on findings related to discussion of the prize-based incentives during exit interviews.

#### Data analysis

Upon recruitment, demographic data, including age, gender identity, race/ethnicity, and relationship status, were collected via a Qualtrics survey and univariate summary statistics were reported. After implementing the prize wheel, we conducted bivariate comparisons between prize categories (silver, blue, pink). Specifically, we used a chi-square test to compare the frequency of each prize selected and to test whether there was a statistically significant difference in prize selection compared with an equal distribution, with a significance set at α = 0.05.

Qualitative data from exit interviews were analyzed via inductive thematic analytic methods.^41^ A subset of transcripts was first open-coded, and an initial codebook was created. This codebook was refined iteratively through double coding by two researchers until acceptable inter-rater reliability (Kappa ≥ .80) was achieved. The remaining transcripts were then coded independently. NVivo software was used to facilitate coding and data management.^43^

## Results

The responses to the pre-implementation prize preference survey generally fall into two groups: older Spanish-speaking adults and younger English-speaking youth, which reflects the diverse perspectives of the community. Overall, both groups demonstrated interest in electronics and fashion items (Table 3). Neither group expressed interest in toys. Older Spanish-speaking adults appeared to prefer beauty and personal care items more than younger English speakers, who preferred home goods and franchise merchandise. These data informed the incentive selection for *PrEP Well*.

We also received qualitative feedback during pre-implementation incentive validation activities (Table 4). Overall, participants expressed overwhelmingly positive support for the incorporation of the wheel and prize incentives. The participants also suggested several prizes to be added, including electronics, apparel, accessories, beauty and art supplies, spiritual items, and outdoor gear.

After the pilot, the wheel and prizes were maintained in the ongoing *PrEP Well* project, and the prize choices were recorded for all participants (Table 5). The participants earned an average of 3.2 (standard deviation: 2.1) spins during their participation in *PrEP Well*. There were a total of 392 spins across all participants, with 58.2% being pink tier spins, 34.2% blue tier spins, and 7.6% silver tier spins.

In each tier, participants were free to choose any available prizes and could choose prizes they had selected previously. For the highest likelihood, lowest price tier (pink) and the moderate likelihood, middle price tier (blue), there was significant difference between which prizes were selected (*p* < 0.0001), suggesting clear preferences for certain items. These preferences did not necessarily correspond to the cost or retail value of the prizes selected. In the lowest likelihood, highest price tier (silver), there was no significant difference in how often participants selected Bluetooth speakers, over the ear headphones, and earbuds (*p* = 0.6703). Originally, apparel items created by a local TGNB-owned business were included in the silver category but they were moved to the blue category because they were not selected when presented in the silver tier.

During qualitative exit interviews, participants expressed approval of the prize wheel. The participants emphasized that the wheel represented an external motivation to improve health, which coupled well with internal motivations to improve health (Table 6). The participants commented positively about the portable chargers, face masks, tuckers, trans pride shoelaces, fidget keychains, socks, estrogen/testosterone necklaces, and candles.

## Discussion

This study demonstrated that creating a TGNB-specific incentive system in partnership with the community built trust and increased the acceptability of *PrEP Well*. Using behavioral economic principles to designate fun components (i.e., a prize wheel), identity affirming prizes, and providing choices with incentive selection led to positive perceptions in *PrEP Well* exit interviews. The high level of acceptability of the prize-based incentive system reflects the importance of community participation in study design and implementation.

The prizes that were integrated with *PrEP Well* reflected the prize selections from the pre-implementation surveys and incentive validation pilot. This also informed targeted purchasing of inventory so that funds were not spent on non-preferred items. The variety of prizes reflected the range of experiences of the TGNB community, including those that faced significant financial instability. For participants, portable chargers, blankets, and scented candles may have provided additional functional benefits. Providing a range of prizes to choose from created an empowerment opportunity as well as an incentive to participate.

Previous research has shown that chance-based incentives alone are not always effective in motivating behavioral change.^28,32,44^ To create effective interventions that lead to sustained behavioral change around HIV prevention behaviors in the TGNB community, public health practitioners should consider creating fun incentive structures that offer participants the opportunity to make choices around gender-affirming incentives. Research has shown that simply giving participants the option to choose from a variety of incentives can restore a sense of internal control and create a more exciting intervention.^34^ Our current findings are consistent and provide examples of ways in which future studies may implement behavioral economic principles to improve HIV prevention behaviors in the TGNB community.

Our qualitative interviews revealed that the participants appreciated the use of trans empowering colors, trans owned businesses, and gender affirming prizes. Qualitative data highlighted the role of external motivators in shaping health behaviors, as well as the nuanced ways these incentives intersected with participants’ personal goals, identities, and community connections. Many participants described struggling with financial insecurity so the addition of prize-based non-monetary incentives to *PrEP Well*, which already included fixed monetary incentives, created a powerful motivator for participation.

While financial incentives can initially attract participants, they may not be as effective for sustained engagement in health-related behaviors.^45^ Previous studies have shown that the effectiveness of interventions decreases significantly after incentives are removed.^28^ We aimed to address this challenge by offering prize-based incentives that would continue to remind participants about their community after their participation in the intervention ended.

One example that reflects these efforts is the tucker panties that were included in the pink tier. These panties can be expensive, difficult to acquire, and vary widely in quality. Through outreach with community partners, we connected with a TGNB-owned small business making high quality tucker panties. These panties were specifically desired by community members and a prize that you would not see in almost any other prize-based interventions.

Although the panties became available in January 2024, 8 months after the implementation of the prize-based incentives, they reached the same levels of selection as prizes that were available for the entire duration. Although the fulfillment timeline was longer than engaging with a large retail entity, we were able to reinvest in the local community and the panties remained one of the most popular prizes. The panties are a memorable product that participants can gain added functional benefits from after study participation has concluded, which may subtly remind them of their experiences in *PrEP Well* and community to positively influence long term PrEP adherence behaviors.

As *PrEP Well* is an ongoing program that continues to evolve based on organizational changes and client preferences, the protocol changed to meet the needs of the community. At one point, our study coordinator noted that some participants felt frustrated to lose spins when they made their best effort to complete a study activity (i.e., completing a doctor visit) but were not able to for reasons out of their control (e.g., delayed insurance authorization). In response, our staff could award a discretionary spin for the participants’ good faith effort. The participants were also allowed to select items from a lower tier category if they preferred it over the higher tier options awarded from their spin. Allowing for this flexibility has led to high levels of acceptability among participants without greatly affecting cost or administrative burden.

### Limitations

This study has several limitations that might hamper its ecological validity. First, the availability of items fluctuates at times due to ordering delays. This was due to the availability of items from vendors, the administrative process of tracking inventory, and the navigation of various institutional procurement processes to pay vendors. Second, because the inventory of items varied, it was not always possible for participants to receive their first-choice prize. Sometimes, a participant would be willing to collect their prize later, when it became available, whereas others would choose a different prize so they could collect it immediately.

Furthermore, *PrEP Well* was conducted at a single TGNB center in Los Angeles, so prize selections may not be generalizable to TGNB individuals elsewhere. However, *PrEP Well* reached a wide array of ages, racial, ethnic, and TGNB identities with consistent results. Finally, because this was a pilot study, a limited number of participants were included in the analyses.

## Conclusions

Future studies should explore the opportunities for recruitment and sustainment that come from creating behavioral economic based incentive systems that are tailored to the specific needs of the target community. This is especially true for members of the TGNB community, who often face stigma and whose needs are frequently overlooked in healthcare settings. Community-driven approaches foster trust, enhance the effectiveness of health interventions, and empower community members to take greater ownership of their health decisions. Additionally, examining the long-term sustainability of behavioral changes initiated through such interventions is critical to understanding the broader impact on HIV prevention efforts. By continuing to center community partnerships and leveraging behavioral economic principles, future interventions can further refine strategies to reduce HIV disparities among TGNB populations.

## Figures and Tables

**Table 1:** Demographics of Pre-exposure prophylaxis (*PrEP*) *Well* participants from April 25, 2023 to January 1, 2025, N = 128

Characteristic	Total (N = 128) N (%)
Assigned sex at birth	
Female	16 (12.5)
Male	110 (85.9)
Intersex	2 (1.6)
Gender identity	
Woman	88 (68.8)
Man	11 (8.6)
Nonbinary	19 (14.8)
Nonbinary trans femme	1 (0.8)
Genderfluid	1 (0.8)
Aliagender	1 (0.8)
Two-spirit	1 (0.8)
Pharaoh	1 (0.8)
None	1 (0.8)
Sexual orientation	
Queer	15 (11.7)
Straight	63 (49.2)
Bisexual	13 (10.2)
Pansexual	22 (17.2)
Gay or lesbian	12 (9.4)
Questioning	2 (1.6)
Omnisexual	1 (0.8)
Race/ethnicity^a^	
American Indian/Alaskan Native	6 (4.7)
Asian	6 (4.7)
Black or African American	28 (21.9)
Hispanic or Latinx	79 (61.7)
Multiracial	23 (18.0)
Other Pacific Islander	3 (2.3)
White non-Hispanic	79 (61.7)
Relationship status	
Single	84 (65.6)
Open relationship	18 (14.1)
Dating/casual relationship	13 (10.2)
Closed relationship	12 (9.4)
Other/prefer not to say	1 (0.8)
On PrEP^b^ at enrollment	25 (19.5)
Age, mean (SD)^c^	31.7 (9.8)
Total number of spins during *PrEP Well*, mean (SD)	3.2 (2.1)

a Not mutually exclusive

b Pre-exposure prophylaxis

c Standard deviation

**Table 2:** Pre-exposure prophylaxis (*PrEP*) *Well* behavioral economic incentive structure.

Participant behavior	Time point	Number of spins earned
Attended information session about *PrEP*^a^ *Well*	Baseline	1 spin
Welcome to *PrEP Well* spin	Baseline	1 spin
Attended initial appointment with PrEP provider	30- or 90-day follow-up	1 spin
Filled PrEP prescription at pharmacy	30- or 90-day follow-up	1 spin
Attended lab/STI^b^ testing visit	30- or 90-day follow-up	1 spin
Urine testing shows PrEP adherence	30- or 90-day follow-up	1 spin
Congratulations on finishing *PrEP Well* program	90	1 spin
Discretionary spin for other positive activities or attempts	Any	1 spin

a Pre-exposure prophylaxis

b Sexually transmitted infection

**Table 3:** Pre-implementation prize preferences

Category	Prize	Total N	Number of selections by Older Spanish speakers (N = 15)	Number of selections by Younger English speakers (N = 9)	Category proportion of total selections
			N (row %)	N (row %)	
Electronics and Tech Accessories N = 37 selections	Earbuds/Headphones	13	5 (38.5)	8 (61.5)	33.9%
	Fitness Tracker	1	0 (0.0)	1 (100.0)	
	iPad	10	5 (50.0)	5 (50.0)	
	Phone Case	2	2 (100.0)	0 (0.0)	
	Pop Socket	2	2 (100.0)	0 (0.0)	
	Portable Charger	6	0 (0.0)	6 (100.0)	
	Smart Watch	3	2 (66.7)	1 (33.3)	
Fashion and Accessories N = 18 selections	Baseball Hat	3	1 (33.3)	2 (66.7)	16.5%
	Fanny Pack	1	0 (0.0)	1 (100)	
	Hoodie/Sweatshirt	5	3 (60.0)	2 (40.0)	
	Necklaces	1	0 (0.0)	1 (100)	
	Rings	3	1 (33.3)	2 (66.7)	
	Socks	5	3 (60.0)	2 (40.0)	
Home Goods N = 12 selections	Crystal Scoop	1	0 (0.0)	1 (100)	11.0%
	Fan	1	0 (0.0)	1 (100)	
	Mugs	5	1 (20.0)	4 (80.0)	
	Sun Catcher	0	0 (0.0)	0 (0.0)	
	Water Bottle	5	0 (0.0)	5 (100.0)	
Toys N = 6 selections	Fidget Toy	2	0 (0.0)	2 (100.0)	5.5%
	Mystery Box	3	2 (66.7)	1 (33.3)	
	Squishies	1	0 (0.0)	1 (100)	
Beauty and Personal Care N = 17 selections	Hair Extensions	5	4 (80.0)	1 (20.0)	15.6%
	Nail Polish	0	0 (0.0)	0 (0.0)	
	Perfume/Cologne	10	9 (90.0)	1 (10.0)	
	Press On Nail Set	2	1 (50.0)	1 (50.0)	
Brand specific prizes N = 19 selections	Batman Brand	3	0 (0.0)	3 (100.0)	17.4%
	Disney Brand	2	2 (100)	0 (0.0)	
	Dragon Ball Brand	2	0 (0.0)	2 (100)	
	Marvel Brand	2	0 (0.0)	2 (100)	
	Pokemon Brand	2	0 (0.0)	2 (100)	
	Sailor Moon Brand	3	0 (0.0)	2 (100)	
	Star Wars Brand	1	0 (0.0)	1 (100)	
	Studio Ghibli Brand	3	0 (0.0)	3 (100)	
	Transformers Brand	1	0 (0.0)	1 (100)	
Total selections		109	43	66	100%

**Table 4:** Themes and example quotes illustrating the perceptions of the prize wheel prior to implementation in Pre-exposure prophylaxis (*PrEP*) *Well*

Theme	Descriptions	Illustrative quotations
Perceptions of prizes	Positive adjectives participants used to describe prizes	“Adorb” “Smart” “Fun”
	Improves accessibility	“I like how accessible [the wheel] is.”
	Anticipated positive effects on participation	“[Adding the new incentives] is definitely the way to go. [The wheel and prizes] will be the best way…to get more participants.” “I think [adding the wheel] will help with participation because more people would come if they c[ould] win something.”
	General positive reception	“I feel like the prizes are really good and [are] worth the time to take the program.”
	Positive perceptions of silver tier	“I really like the silver tier prizes cause it’s stuff we would need that we can’t get.”

**Table 5:** Comparison of top prize selections for each category, from April 25, 2023 to January 1, 2025

Prize category	Prize	Times selected N (row %)	Chi Square p-value^a^
Silver N = 30 spins	Bluetooth speaker	8 (26.7)	0.6703
	Over-the-ear headphones	10 (33,3)	
	Earbuds	12 (40.0)	
Blue N = 134 spins	Portable charger	46 (34.3)	<0.0001
	Trans pride or solid color blanket	35 (19.4)	
	Trans pride attire (e.g., t-shirt, bracelet, necklace)	18 (13.4)	
	Stickers	9 (6.7)	
	Stealth Bros Dopp kit	8 (6.0)	
	Estrogen or testosterone necklace	8 (6.0)	
	Mars products (e.g., fanny pack, mug)	6 (4.5)	
	Camping hammock	4 (3.0)	
Pink N = 228 spins	Candle	70 (30.7)	<0.0001
	Fidget toys	37 (16.2)	
	Face mask, eye mask, clay mask	32 (14.0)	
	Tucker panties	32 (14.0)	
	Pill organizer	24 (10.5)	
	Mars branded items (e.g., socks, ornament, art prints, pop socket)	14 (6.4)	
	Trans pride shoelaces	12 (5.3)	
	Lava lamp pen	7 (3.1)	

a Compared to equal distribution of prize options

**Table 6:** Themes and example quotes illustrating the perceptions of the prize wheel after implementation in *PrEP Well*

Theme	Descriptions	Illustrative quotations
Perceptions of wheel	Positive perceptions of the prize wheel	“[The wheel] turns a stressful time into something fun, something that makes you want to come.” “Nobody does [a prize wheel] in other places.” “Not all of us had the childhood we wanted…so it’s kind of nice when we do get to experience something that’s… just fun and [spinning a prize wheel is] something that you’d do as a kid.”
Prize specific feedback	Appreciation of trans pride themed items	“Our identity is very important to all of us and especially in the world we live in.”
	Positive perception of portable charger	I got the battery charger on my first time. And I still get teared up. It’s pretty cool.”
	Positive perception of blanket	“The truth is that even though the [prizes] look very small, they mean a lot because it’s really a basic thing. [Winning the blanket] did help me at one point for the cold weather or to cover [my]self with something.”
	Positive perceptions of noise cancelling headphones	“I really liked the earphones because I have social anxiety. So me going home from getting those buds and them being in my ear, ignoring the world, them were like a lifesaver.” “Every time I use [my headphones], I think about [PrEP Well]…[I] see why you should do things like [PrEP Well]. Because good things could come from it.”

## Data Availability

The datasets generated and/or analysed during the current study are not publicly available because participants of this study did not give written consent for their data to be shared publicly but are available from the corresponding author on reasonable request.

## Abbreviations

PrEP: Pre-exposure prophylaxis
TGNB: Transgender and gender nonbinary
LGBTQ: Lesbian, gay, bisexual, transgender, and queer
